# Development of Multiscale Transcriptional Regulatory Network in Esophageal Cancer Based on Integrated Analysis

**DOI:** 10.1155/2020/5603958

**Published:** 2020-08-12

**Authors:** Zihao Xu, Zilong Wu, Jingtao Zhang, Ruihao Zhou, Jiane Wu, Bentong Yu

**Affiliations:** ^1^Department of Thoracic Surgery, The First Affiliated Hospital of Nanchang University, Nanchang, Jiangxi 330006, China; ^2^School of Public Health, Nanchang University, Nanchang, Jiangxi 330006, China; ^3^The First Clinical Medical College, Nanchang University, Nanchang, Jiangxi 330006, China; ^4^Department of Pain Management, West China Hospital, Sichuan University, Chengdu, Sichuan 610000, China; ^5^Department of Anesthesiology, Hongdu Traditional Chinese Medicine Hospital of Nanchang, Nanchang, Jiangxi 330006, China

## Abstract

**Objective:**

To explore multiscale integrated analysis methods in identifying key regulators of esophageal cancer (ESCA).

**Methods:**

We downloaded the ESCA dataset from The Cancer Genome Atlas (TCGA) database, which contained RNA-seq data, miRNA-seq data, methylation data, and clinical phenotype information. Then, we combined ESCA-related genes from the NCBI-GENE and OMIM databases and RNA-seq dataset from TCGA to analyze differentially expressed genes (DEGs). Meanwhile, differentially expressed miRNAs (DEmiRNAs) and genes with differential methylation levels were identified. The pivot–module pairs were established using the RAID v2.0 database and TRRUST v2 database. Next, the multifactor-regulated functional network was constructed based on the above information. Additionally, gene corresponding targeted drug information was obtained from the DrugBank database. Moreover, we further screened regulators by assessing their diagnostic value and prognostic value, especially the value of distinguishing patients at TNM I stage from normal patients. In addition, the external database from the Gene Expression Omnibus (GEO) database was used for validation. Lastly, gene set enrichment analysis (GSEA) was performed to explore the potential biological functions of key regulators.

**Results:**

Our study indicated that CXCL8, CYP2C8, and E2F1 had excellent diagnostic and prognostic values, which may be potential regulators of ESCA. At the same time, the good early diagnosis ability of the three regulators also provided new insights for the diagnosis and early treatment of ESCA patients.

**Conclusion:**

We develop a multiscale integrated analysis and suggest that CXCL8, CYP2C8, and E2F1 are promising regulators with good diagnostic and prognostic values in ESCA.

## 1. Introduction

Esophageal cancer (ESCA) ranks the seventh among all cancer incidences and the sixth in mortality overall with a 5-year survival rate of about 19% [1, 2]. Endoscopy is emerging as a frontline treatment option for ESCA patients at the early stage with promising results [3, 4]. Unfortunately, most patients with ESCA are detected at an advanced stage [5]. Therefore, despite the development of endoscopic therapy, neoadjuvant chemoradiotherapy, and surgery, the prognosis of patients with ESCA is still not optimistic [6–8]. The identification of new biomarkers is critical to improve the early diagnosis and treatment of ESCA.

Previous studies have developed a mass of methods to explore the valuable factors in ESCA. For instance, Xue et al. searched for key factors by constructing a specific competitive endogenous RNA network in ESCA [9]. The 8-mRNA-based risk score model developed by Cai et al. successfully predicted the survival of ESCA [10]. Ushiku et al. confirmed that promoter DNA hypermethylation of CDO1 could be an independent prognostic factor in ESCC [11]. However, most studies are built on single-dimensional analysis, which is difficult to get convincing results, especially in cancers with a small sample size such as ESCA. Therefore, it is particularly crucial to explore multiscale integrated analysis methods in identifying key regulators of cancer.

**Figure 1:**
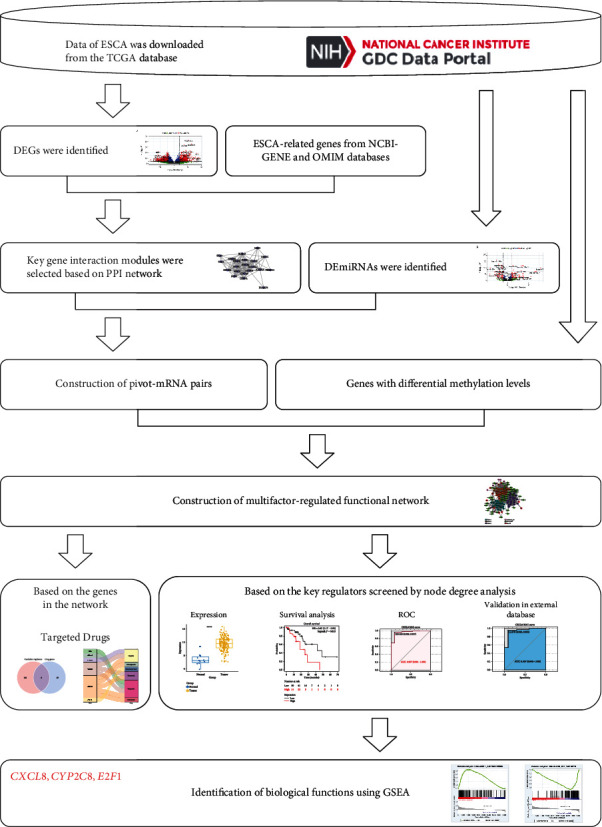
Flowchart of identification of key regulators based on multiscale integrated analysis.

The Cancer Genome Atlas (TCGA) database contains a wealth of publicly available datasets, providing multiple types of genomic data and clinical information [12]. In this study, we downloaded RNA-seq data, miRNA-seq data, methylation data, and clinical phenotype information from the TCGA database. The construction of a multifactor-regulated functional network was then performed through differential expression analysis, node degree analysis, and pivot analysis. Next, we obtained key regulators and further explored their targeted drugs. Furthermore, the diagnostic and prognostic values of the key regulators for ESCA patients were considered as the means of further screening. Ultimately, gene set enrichment analysis (GSEA) was used to clarify the biological functions of the key regulators. Overall, our study offers a novel method and insight for the identification of key regulators in ESCA based on multiscale integrated analysis (Figure 1).

## 2. Materials and Methods

### 2.1. Data Sources and Preprocessing

Data of ESCA was obtained from the TCGA database, including gene expression data of 142 ESCA and 9 control samples, methylation chip data of 162 ESCA and 14 normal samples, miRNA expression profile data of 161 ESCA and 11 normal samples, and clinical phenotype data. 505 genes related to ESCA from the NCBI-GENE database [13] (http://www.ncbi.nlm.nih.gov/gene) and OMIM database [14] (http://omim.org/) were selected for analysis. Gene expression data of GSE53625 which contained 179 tumor samples and 179 paired normal samples was downloaded from the Gene Expression Omnibus (GEO) database [15] for validation.

### 2.2. Differential Expression Analyses

Based on RNA-seq data and miRNA-seq data, differential expression analysis was performed by DESeq2 R package [16]. Based on the methylation data, differential expression analysis was performed by limma R package [17]. Furthermore, differential methylation sites were mapped to corresponding genes. Genes with ultrahigh and ultralow methylation levels were then screened out by annotating these differential sites. The pheatmap R package [18] was used to visualize the heatmap.

### 2.3. Construction of Coexpression Network

The STRING database [19] (https://string-db.org/) was used to construct a protein–protein interaction (PPI) network, and the interaction network of candidate genes was selected based on score > 900. Visualization was performed using Cytoscape software [20]. Furthermore, interactive network clustering analysis on candidate gene interaction networks was performed using ClusterONE plugin in Cytoscape software.

### 2.4. Kyoto Encyclopedia of Genes and Genomes (KEGG) and Gene Ontology (GO) Enrichment Analyses

Functional enrichment analyses, including Gene Ontology (GO) comprising biological process (BP) and Kyoto Encyclopedia of Genes and Genomes (KEGG), were performed on key gene interaction modules using enrichGO and enrichKEGG functions of clusterProfiler R package [21]. The *P* value < 0.05 adjusted by the Benjamini and Hochberg method was deemed to be statistically significant.

### 2.5. Identification of ncRNAs and TFs Based on Pivot Analysis

Pivot analysis refers to screening out strongly related genes of module genes by hypergeometric test, and the pivot nodes mean (i) at least two interaction pairs with module genes and (ii) significant interaction between the node and each module. Moreover, the pivot nodes with *P* value < 0.05 were considered significant.

Based on the identification criteria of pivot nodes, using the ncRNA–mRNA interaction relationship included in the RAID v2.0 database [22] (http://www.rna-society.org/raid2/index.html) as the interaction background, the interaction pairs of ncRNAs and module genes were established. Similarly, using the TF–mRNA regulation relationship included in the TRRUST v2 database [23] (https://www.grnpedia.org/trrust/) as the interaction background, the interaction pairs of TFs and module genes were established.

### 2.6. Construction of Multifactor-Regulated Functional Network

Based on the establishment of network modules, DEmiRNAs, differentially expressed methylation level genes, and significantly related TFs and ncRNAs screened by pivot analysis, the multifactor-regulated functional network was established. Through the analysis of node degrees and functional enrichment analysis, further screening for possible key genes related to ESCA was performed. Besides, the information of the drugs corresponding to the candidate regulators was obtained from the DrugBank database [24].

### 2.7. Identification of Key Regulators

ROC curve analysis was performed using the pROC R package [25], assessing the diagnostic value of the key genes for ESCA and TNM I stage of cancer. Genes with the area under the ROC curve (AUC) > 0.7 were considered to be diagnosis-related genes for ESCA patients. The Kaplan-Meier analysis was performed using the Kaplan-Meier plotter [26] (http://kmplot.com/analysis/), and the boxplots were used to visualize the expression levels of prognosis-related genes between ESCA and normal samples. The *P* value < 0.05 was considered to be statistically significant.

### 2.8. GSEA of Key Regulators

To explore the potential biological functions of the key regulators, GSEA was performed using GSEA4.0.3 software. The samples were divided into high and low groups using the median of the expression levels of key regulators as the cutoff value. The hall mark gene set (h.all.v7.1.symbols.gmt) was downloaded from the MSigDB database [27]. The gene sets with *P* value < 0.05 after 1000 permutations were considered significantly enriched gene sets.

## 3. Results

### 3.1. Identification of DEGs and DEmiRNAs

Based on downloaded RNA-seq data, differential expression analysis was performed using the DESeq2 R package. According to the threshold (∣log2FC | >2 and *P* value < 0.0001), 1341 DEGs (733 upregulated and 608 downregulated) were obtained (Figure S1). Similarly, DEmiRNAs were analyzed using ∣log2FC | >1 and *P* value < 0.01 as the cutoff criteria based on the miRNA-Seq data, 95 DEmiRNAs (51 upregulated and 36 downregulated) were identified (Figure S2).

### 3.2. Identification of Key Gene Interaction Modules

**Figure 2:**
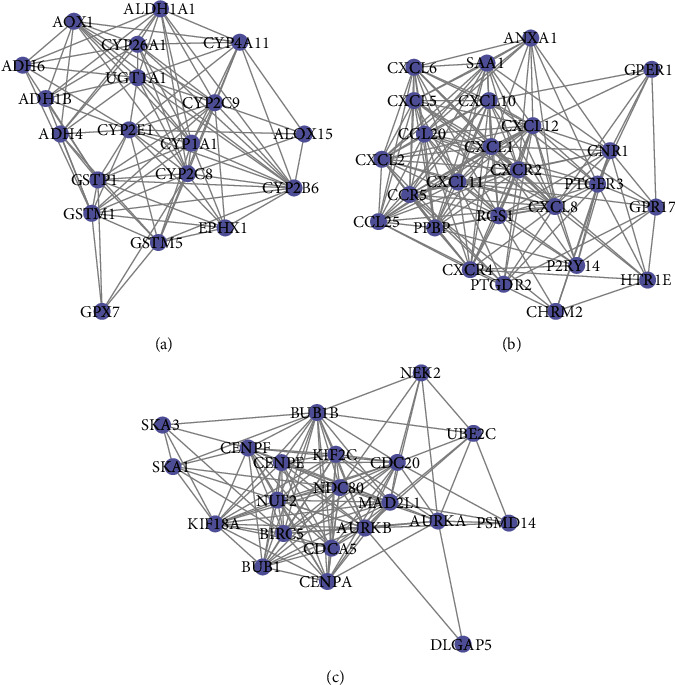
Top 3 related gene interaction network modules selected by ClusterONE plugin. (a) Cluster 1. (b) Cluster 2. (c) Cluster 3.

The candidate gene set contained 1793 genes was obtained by combining the DEGs with the ESCA-related genes from the NCBI-GENE and OMIM databases. Based on the candidate gene set, a PPI interaction network was constructed, including a total of 2785 edges and 1793 points (Figure S3). Next, the ClusterONE plugin was used to mine modules on the network and filter the modules that interact significantly (*P* < 0.05); a total of 17 modules were obtained (Supplementary Table 1). Then, the top 3 related gene interaction network modules were selected for further analysis, named key gene interaction modules (Figures 2(a)–2(c)).

To further explore whether key gene interaction modules have the ability to guide the stage of ESCA, we visualized the expression levels of 65 genes in the modules. Moreover, the 65 genes could distinguish tumor samples from normal samples, and cancer stage ii and iii could be well clustered together. The genes of the three modules also showed unique expression patterns. The genes in each module were well clustered, especially Cluster 3 (Figure S4).

### 3.3. Functional and Pathway Enrichment Analyses of Key Gene Interaction Modules

**Figure 3:**
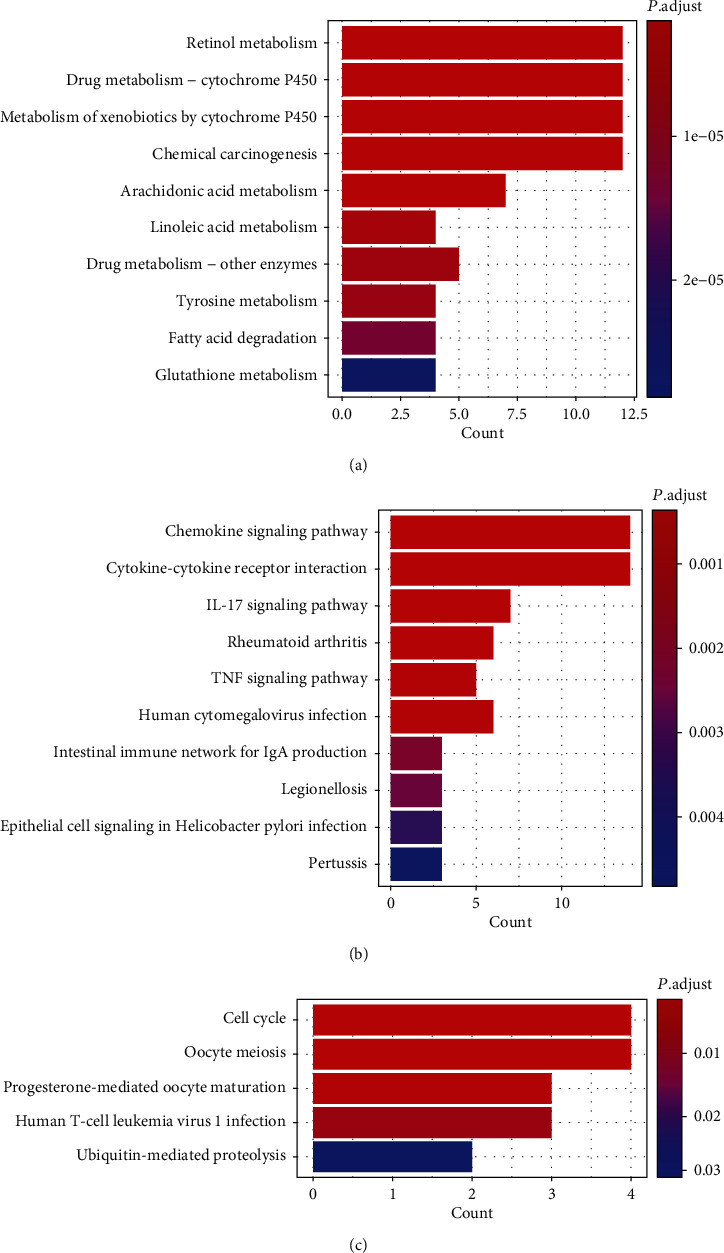
Functional and pathway enrichment analyses of key gene interaction modules. (a) KEGG and GO enrichment of Cluster 1. (b) KEGG and GO enrichment of Cluster 2. (c) KEGG and GO enrichment of Cluster 3. (*P* value < 0.05; the *Y*-axis of the figure represents GO terms and KEGG pathways, and the *X*-axis represents the number of genes per GO term or KEGG pathway).

Furthermore, to elucidate key gene interaction modules involved in the development of ESCA, functional and pathway enrichment analyses were performed using the ClusterProfiler package in R. As the result showed, Cluster 1 was enriched in pathways such as retinol metabolism and drug metabolism, and Cluster 2 was enriched in chemokine, IL-17, and TNF signaling pathway, while Cluster 3 participated in biological processes such as cell cycle (Figures 3(a)-–3(c)).

### 3.4. Regulation Relationships of ncRNAs and TFs on Key Gene Interaction Modules

Based on the 51913 pairs of ncRNA–mRNA interactions included in the RAID v2.0 database, the pivot nodes (ncRNAs) that regulated the key gene interaction modules were identified; with *P* value <0.01 as the screening criteria, a total of 43 ncRNA–module interaction pairs were screened (Supplementary Table 2). Similarly, based on the 9396 human TF–mRNA interactions included in the TRRUST v2 database, a total of 11 TF–module interaction pairs were screened by using *P* value < 0.005 as the screening criteria (Supplementary Table 3).

### 3.5. Identification of Genes with Differential Methylation Levels

Based on the ESCA methylation data, according to the threshold (∣log2FC | >2 and *P* value < 0.0001), 11025 differential methylation sites were obtained. Further, these sites were mapped to corresponding genes, 202 genes of ∣log2FC | >2 were identified (10 with high methylation level and 192 with low methylation level).

### 3.6. Multifactor-Regulated Functional Network Construction and Mining

**Figure 4:**
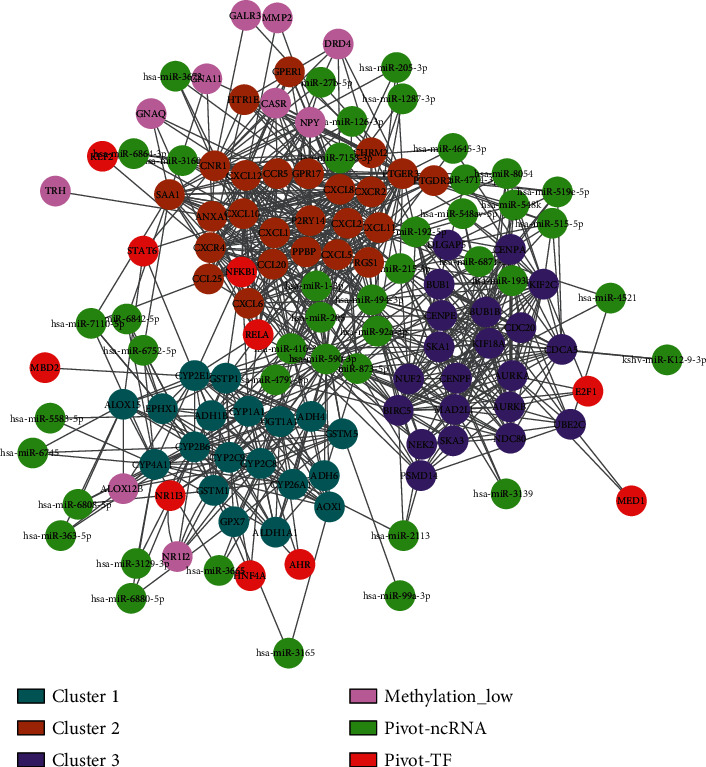
Multifactor-regulated functional network.

**Figure 5:**
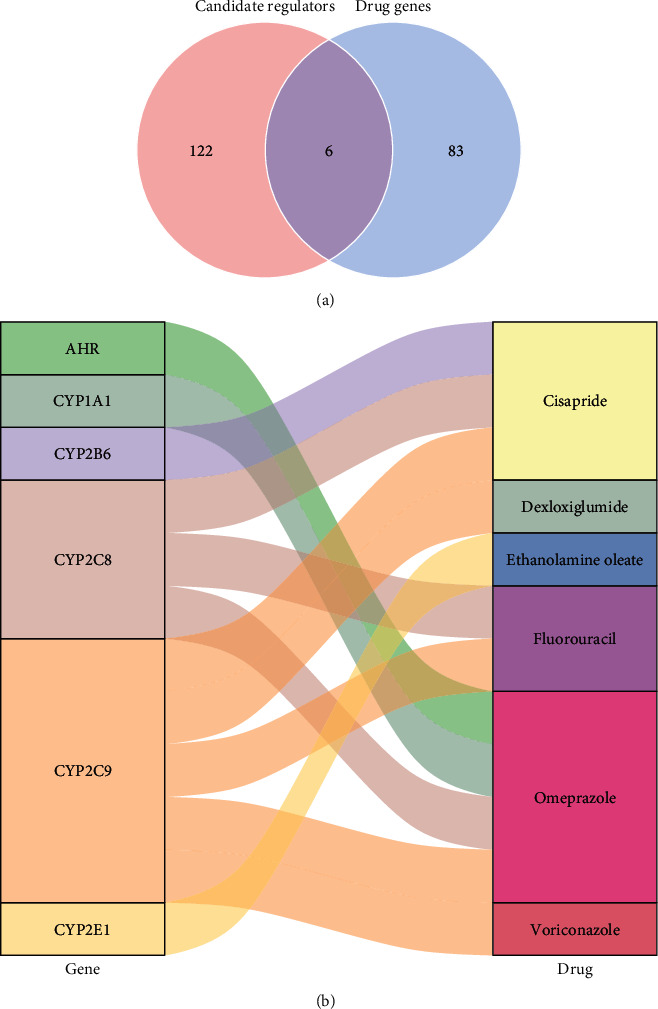
Exploration of gene-targeted drugs in multifactor-regulated functional network. (a) Intersection of candidate regulators with ESCA-related genes from the DrugBank database. (b) Targeting drugs corresponding to intersection genes.

Based on the 43 ncRNAs and 11 TFs obtained from the pivot analysis above, combined with DEmiRNAs and genes with differential methylation levels, 128 candidate regulators were used to construct the multifactor-regulated functional network according to protein–protein interaction and protein–ncRNA interaction information (Figure 4). Then, 28 drugs related to ESCA and 89 corresponding drug genes were retrieved from the DrugBank database. Next, 6 genes were obtained from the intersection of drug genes and the candidate regulators, and the corresponding drugs were cisapride, omeprazole, dexloxiglumide, fluorouracil, voriconazole, and ethanolamine oleate (Figures 5(a) and 5(b)). Moreover, based on the calculation of the degree of each node (Supplementary Table 4), the top 3 nodes of each type of regulators were selected as the key regulators of ESCA (Table 1).

### 3.7. Identification of Key Regulators and Verification Based on an External Database

**Figure 6:**
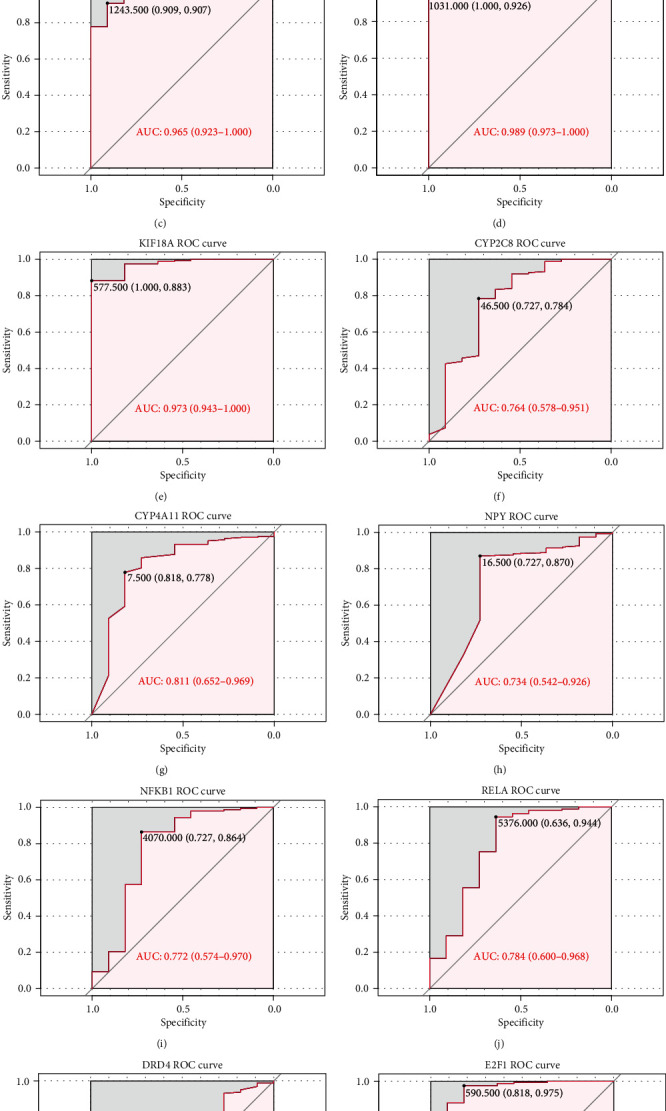
The diagnostic value of key regulators in distinguishing ESCA patients from normal controls. (a) CXCL8: AUC 0.957. (b) PTGER3: AUC 0.704. (c) MAD2L1: AUC 0.965. (d) BIRC5: AUC 0.989. (e) KIF18A: AUC 0.973. (f) CYP2C8: AUC 0.764. (g) CYP4A11: AUC 0.811. (h) NPY: AUC 0.734. (i) NFKB1: AUC 0.772. (j) RELA: AUC 0.784. (k) DRD4: AUC 0.730. (l) E2F1: AUC 0.953.

**Figure 7:**
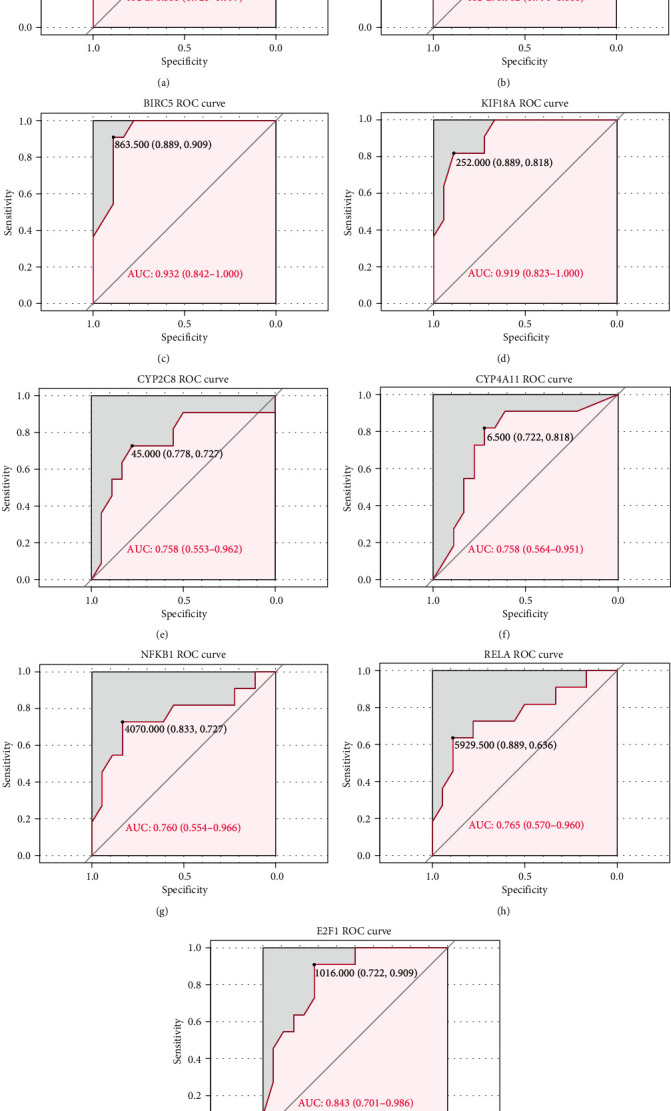
The diagnostic value of key regulators in distinguishing ESCA patients at TNM I stage from normal controls. (a) CXCL8: AUC 0.861. (b) MAD2L1: AUC 0.902. (c) BIRC5: AUC 0.932. (d) KIF18A: AUC 0.919. (e) CYP2C8: AUC 0.758. (f) CYP4A11: AUC 0.758. (g) NFKB1: AUC 0.760. (h) RELA: AUC 0.765. (i) E2F1: AUC 0.843.

**Figure 8:**
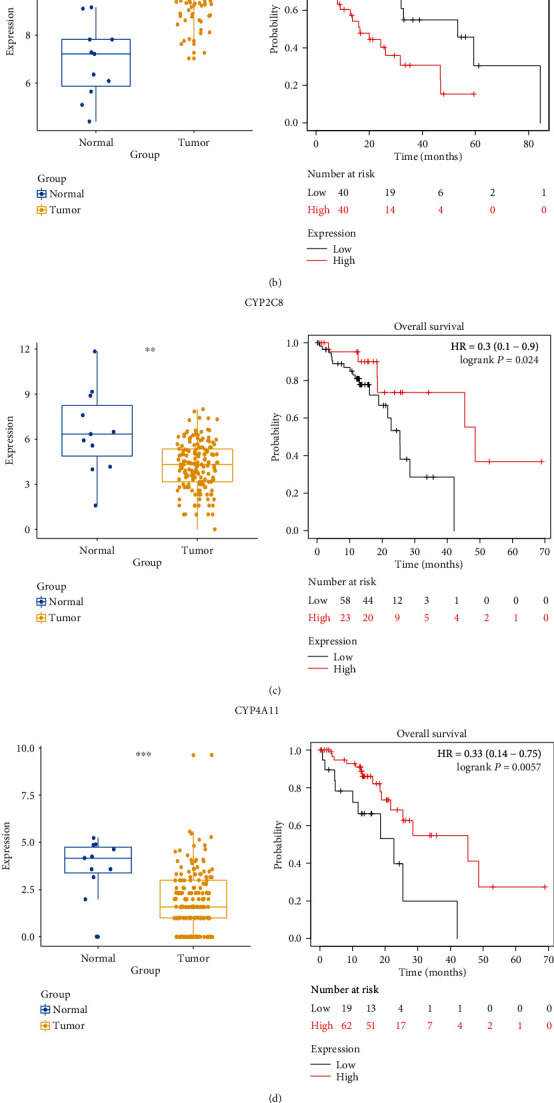
The boxplot of expression levels and survival curves for key regulators associated with overall survival in ESCA. (a) CXCL8. (b) KIF18A. (c) CYP2C8. (d) CYP4A11. (e) E2F1.

Firstly, based on the key regulators identified, ROC curve analysis was performed to evaluate the diagnostic value in distinguishing ESCA patients from normal controls (Figure 6). In order to further test the early diagnosis ability of key regulators, regulators with AUC > 0.7 were selected to analyze the diagnostic performance in distinguishing ESCA patients at TNM I stage from normal individuals (Figure 7). CXCL8, MAD2L1, BIRC5, KIF18A, CYP2C8, CYP4A11, NFKB1, RELA, and E2F1 showed excellent diagnostic value. Furthermore, through analysis of gene expression levels in tumor and normal samples and survival analysis, CXCL8, KIF18A, and E2F1 showed high expression and poor prognosis, which indicated that they may play a role in promoting ESCA progression. Conversely, CYP2C8 and CYP4A11 showed high expression and good prognosis, suggesting a potential protective role against cancer pathogenicity (Figure 8). Finally, the 5 regulators also showed good diagnostic performance in the validation dataset from the GEO database (Figure S5). Notably, CXCL8, CYP2C8, and E2F1 also had a good value in distinguishing patients at TNM I stage from normal controls, which might be promising biomarkers for early diagnosis of ESCA (Figure S6).

### 3.8. GSEA of CXCL8, CYP2C8, and E2F1

**Figure 9:**
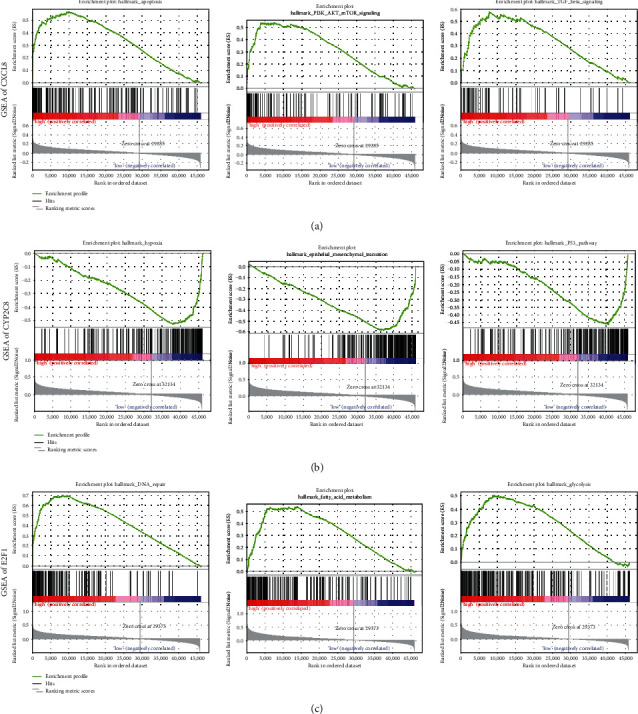
Gene set enrichment analysis of CXCL8, CYP2C8, and E2F1. (a) CXCL8, (b) CYP2C8, and (c) E2F1 (*P* value < 0.05).

To clarify the potential biological functions of CXCL8, CYP2C8, and E2F1, GSEA was performed. The results suggested that the key regulators were significantly associated with cancer-related pathways. For instance, the high expression level of CXCL8 was associated with apoptosis, P13K/AKT/mTOR signaling, and TGF beta signaling (Figure 9(a)). The low expression level of CYP2C8 was mainly involved in hypoxia, epithelial mesenchymal transition, and P53 pathway (Figure 9(b)). Additionally, highly expressed E2F1 was correlated with DNA repair, fatty acid metabolism, and glycolysis (Figure 9(c)).

## 4. Discussion

In this study, we identified DEGs, DEmiRNAs, and genes with differential methylation levels based on the data from TCGA. Then, PPI network was constructed by using the candidate gene set. After the module screening of the coexpression network, the key gene interaction modules containing 65 genes was obtained. The 43 ncRNA–module pairs and 11 TF–module pairs were established. Besides, the multifactor-regulated functional network containing 128 candidate regulators was constructed base on the above data. Through the screening of the node degree, we got the top 3 nodes of each type of regulators. Subsequently, CXCL8, CYP2C8, and E2F1 were selected with satisfactory diagnosis and prognostic value in ESCA. Finally, GSEA results showed the potential biological functions of these three key regulators.

Heatmap cluster analysis showed that 65 genes of the key gene interaction modules might guide the stage of ESCA. Meanwhile, the key gene interaction modules were mainly enriched in the cancer-related pathway and biological process, such as retinol metabolism signaling pathways, chemokine signaling pathways, and cell cycle. Chemokine-neutralizing antibodies might significantly attenuate the effect of CAF on hepatocellular carcinoma metastasis [28]. More notably, the key gene interaction modules contained a large number of CXC family genes. As a family of cytokines, CXC chemokines had been confirmed to be pleiotropic in regulating tumor-associated angiogenesis and cancer cell metastasis [29]. Research by Yasumoto et al. proved that the CXCR4/CXC12 axis plays an important role in the development of peritoneal carcinomatosis from gastric carcinoma [30].

In order to analyze as many potential regulators as possible, we constructed pivot-mRNA pairs based on the key gene interaction modules. Moreover, the multifactor-regulated functional network containing 128 candidate regulators was constructed. DrugBank is a freely available database that combines detailed drug data with comprehensive drug-target information [31]. By searching for ESCA-related genes and drugs and combining 128 candidate regulators, 6 genes and their targeted drugs were screened. It was worth noting that CYP2C8 as the final selected regulator was related to cisapride, omeprazole, and fluorouracil. Previous research had indicated that CYP2C8 could be used as a reliable predictor of drug response [32]. Among the drugs obtained, omeprazole may yield valuable insight into clinical treatment of Barrett's esophagus progression [33]. Also, the trimodality therapy with fluorouracil as a standard treatment for patients with ESCA reflects a long-term survival advantage [34].

Among the regulators that were not finally selected, there were also some well-known factors that had been declared in cancer. Silencing KIF18A induced apoptosis in lung adenocarcinoma cells and blocked the cell cycle at G2/M phase, while overexpression of KIF18A might promote cell proliferation and inhibit apoptosis [35]. Moreover, Kim et al. suggested that CYP4A11 expression was a potential poor prognostic factor of renal cell carcinoma [36]. After assessing the diagnosis and prognostic values of identified key regulators in both the TCGA and GEO verification datasets, CXCL8, CYP2C8, and E2F1 were selected, which were reported as potential biomarkers in some cancers. Overexpression of CXCL8 was related to tumor progression, metastasis, higher preoperative levels of proinflammatory cytokines, CRP, activation of exogenous coagulation factors, and poor prognosis in esophageal squamous cell carcinoma patients [37]. Moreover, the CYP2C8 gene expression level was confirmed as a potential prognostic marker for hepatocellular carcinoma after hepatectomy [38]. E2F1 could induce TINCR transcriptional activity and accelerated the progression of gastric cancer by activating the TINCR/STAU1/CDKN2B signaling axis [39]. It was more noteworthy that these genes showed good early diagnosis ability, which was of great significance for the timely diagnosis of ESCA patients. Identification of biomarkers for early diagnosis had always been the focus of ESCA research. Plasma POU3F3 was confirmed as a potential biomarker for diagnosis of ESCC, and the combination of POU3F3 and SCCA was efficient for early tumor screening [40]. Nonetheless, there were few biomarkers with early diagnostic value that have been proven in ESCA. Our results provided directions for further experimental and clinical validation.

Furthermore, we performed GSEA to clarify the potential biological functions of CXCL8, CYP2C8, and E2F1. The results indicated that some crucial cancer-related pathways were related to the high expression of CXCL8 and E2F1 and the low expression of CYP2C8, which was consistent with the expression level results and prognostic results. Targeting apoptosis in cancer is feasible, and the exploration of treatment strategies aimed at enhancing apoptosis remained an essential direction in tumor treatment [41]. P13K/AKT/mTOR signaling had been confirmed to play an important role in proliferation, migration, invasion, and chemotherapy resistance [42]. TGF beta signaling, as a widely concerned pathway in cancer, affected tumor cells and the tumor microenvironment, accordingly affecting cancer development [43]. Additionally, epithelial mesenchymal transition could be induced by TGF-*β*, in which epithelial cells acquired mesenchymal phenotype, leading to enhanced motility and invasion in cancer progression [44]. Genes involved in DNA repair responses presented various types of mutations and abnormal expressions in cancer cells, which might cause genomic instability and promote cancer progression [45]. Moreover, glycolysis, the process of conversion of glucose into pyruvate followed by lactate production, played a crucial role in energy metabolism. Therefore, glycolysis could be used as a target for cancer therapy according to the general situation of altered energy metabolism in tumor progression [46].

In conclusion, our study provides a novel method and insight for exploring key regulators in cancer. Differential expression analysis, node degree analysis, pivot analysis, and the construction of a multifactor-regulated functional network are fully taken into account. Based on such multidimensional comprehensive analysis, more interesting results will be explored.

## 5. Conclusion

This study focuses on multiscale integrated analysis and suggests that CXCL8, CYP2C8, and E2F1 are promising regulators in ESCA.

## Figures and Tables

**Table 1:** Key regulators.

Group	Regulators
Cluster 1	CYP2B6, CYP2C8, and CYP4A11
Cluster 2	CXCL8, PTGER3, and CXCL12
Cluster 3	MAD2L1, BIRC5, and KIF18A
Methylation_low	NPY, CASR, and DRD4
Pivot-ncRNA	Hsa-miR-590-3p, hsa-miR-494-3p, and hsa-miR-410-3p
Pivot-TF	NFKB1, RELA, and E2F1

## Data Availability

The datasets analyzed in this study are available in The Cancer Genome Atlas (TCGA) and Gene Expression Omnibus (GEO).

## Supplementary Materials

Supplementary Materials Supplementary Materials Figure S1 (A) volcano plot of RNA-seq data between ESCA tissues and normal tissues. (B) Heatmap of DEGs with log2 (x +1) scale. Figure S2: (A) volcano plot of miRNA-seq data between ESCA tissues and normal tissues. (B) Heatmap of DEmiRNAs with log2 (x +1) scale. Figure S3: PPI network based on candidate gene set. Figure S4: heatmap showing the staging capabilities of key gene interaction modules. Figure S5: the diagnostic value of key regulators in distinguishing ESCA patients from normal controls based on GSE53625. (A) CXCL8: AUC 0.923. (B) KIF18A: AUC 0.899. (C) CYP2C8: AUC 0.922. (D) CYP4A11: AUC 0.738. (E) E2F1: AUC 0.849. Figure S6: the diagnostic value of key regulators in distinguishing ESCA patients at TNM I stage from normal controls based on GSE53625. (A) CXCL8: AUC 0.937. (B) CYP2C8: AUC 0.810. (C) E2F1: AUC 0.841. Supplementary Table 1: candidate gene interaction networks. Supplementary Table 2: pivot (ncRNA)-module pairs. Supplementary Table 3: pivot (TF)-module pairs. Supplementary Table 4: candidate regulators
