# Supplementary material for: Development of Multiscale Transcriptional Regulatory Network in Esophageal Cancer Based on Integrated Analysis
Source: Biomed Res Int. 2020 Aug 12;2020:5603958. doi: 10.1155/2020/5603958 (PMC7441423; doi:10.1155/2020/5603958)
Supplement: Supplementary Materials — Figure S1 (A) volcano plot of RNA-seq data between ESCA tissues and normal tissues. (B) Heatmap of DEGs with log2 (x +1) scale. Figure S2: (A) volcano plot of miRNA-seq data between ESCA tissues and normal tissues. (B) Heatmap of DEmiRNAs with log2 (x +1) scale. Figure S3: PPI network based on candidate gene set. Figure S4: heatmap showing the staging capabilities of key gene interaction modules. Figure S5: the diagnostic value of key regulators in distinguishing ESCA patients from normal controls based on GSE53625. (A) CXCL8: AUC 0.923. (B) KIF18A: AUC 0.899. (C) CYP2C8: AUC 0.922. (D) CYP4A11: AUC 0.738. (E) E2F1: AUC 0.849. Figure S6: the diagnostic value of key regulators in distinguishing ESCA patients at TNM I stage from normal controls based on GSE53625. (A) CXCL8: AUC 0.937. (B) CYP2C8: AUC 0.810. (C) E2F1: AUC 0.841. Supplementary Table 1: candidate gene interaction networks. Supplementary Table 2: pivot (ncRNA)-module pairs. Supplementary Table 3: pivot (TF)-module pairs. Supplementary Table 4: candidate regulators [file 5603958.f1.zip › Supplementary Table1.docx]

Supplementary Table 1: Candidate gene interaction networks

| Cluster | Size | P-value |
| --- | --- | --- |
| 1 | 19 | 5.71E-08 |
| 2 | 25 | 1.25E-07 |
| 3 | 21 | 1.16E-05 |
| 4 | 7 | 0.001118 |
| 5 | 6 | 0.001134 |
| 6 | 7 | 0.001194 |
| 7 | 10 | 0.001474 |
| 8 | 5 | 0.003251 |
| 9 | 6 | 0.003321 |
| 10 | 5 | 0.004618 |
| 11 | 10 | 0.007208 |
| 12 | 4 | 0.010104 |
| 13 | 3 | 0.029673 |
| 14 | 3 | 0.029673 |
| 15 | 3 | 0.029673 |
| 16 | 7 | 0.032404 |
| 17 | 6 | 0.043491 |
