# Supplementary material for: Development of Multiscale Transcriptional Regulatory Network in Esophageal Cancer Based on Integrated Analysis
Source: Biomed Res Int. 2020 Aug 12;2020:5603958. doi: 10.1155/2020/5603958 (PMC7441423; doi:10.1155/2020/5603958)
Supplement: Supplementary Materials — Figure S1 (A) volcano plot of RNA-seq data between ESCA tissues and normal tissues. (B) Heatmap of DEGs with log2 (x +1) scale. Figure S2: (A) volcano plot of miRNA-seq data between ESCA tissues and normal tissues. (B) Heatmap of DEmiRNAs with log2 (x +1) scale. Figure S3: PPI network based on candidate gene set. Figure S4: heatmap showing the staging capabilities of key gene interaction modules. Figure S5: the diagnostic value of key regulators in distinguishing ESCA patients from normal controls based on GSE53625. (A) CXCL8: AUC 0.923. (B) KIF18A: AUC 0.899. (C) CYP2C8: AUC 0.922. (D) CYP4A11: AUC 0.738. (E) E2F1: AUC 0.849. Figure S6: the diagnostic value of key regulators in distinguishing ESCA patients at TNM I stage from normal controls based on GSE53625. (A) CXCL8: AUC 0.937. (B) CYP2C8: AUC 0.810. (C) E2F1: AUC 0.841. Supplementary Table 1: candidate gene interaction networks. Supplementary Table 2: pivot (ncRNA)-module pairs. Supplementary Table 3: pivot (TF)-module pairs. Supplementary Table 4: candidate regulators [file 5603958.f1.zip › Supplementary Table2.docx]

Supplementary Table 2: Pivot (ncRNA) - Module pairs

| Cluster | ncRNA | Connection | P-value |
| --- | --- | --- | --- |
| 1 | hsa-miR-4797-5p | 3 | 0.001588 |
| 1 | hsa-miR-3665 | 3 | 0.003563 |
| 1 | hsa-miR-6880-5p | 3 | 0.00414 |
| 1 | hsa-miR-6745 | 3 | 0.005455 |
| 1 | hsa-miR-363-5p | 3 | 0.005455 |
| 1 | hsa-miR-6752-5p | 3 | 0.005819 |
| 1 | hsa-miR-6842-5p | 3 | 0.005819 |
| 1 | hsa-miR-3165 | 2 | 0.005831 |
| 1 | hsa-miR-6808-5p | 4 | 0.0082 |
| 1 | hsa-miR-99a-3p | 1 | 0.008277 |
| 1 | hsa-miR-7110-5p | 3 | 0.008768 |
| 1 | hsa-miR-590-3p | 11 | 0.009035 |
| 1 | hsa-miR-5583-5p | 2 | 0.009592 |
| 1 | hsa-miR-3129-3p | 2 | 0.009592 |
| 2 | hsa-miR-1-3p | 14 | 0.000188 |
| 2 | hsa-miR-3160-5p | 5 | 0.000696 |
| 2 | hsa-miR-27b-5p | 3 | 0.001183 |
| 2 | hsa-miR-1287-3p | 3 | 0.001905 |
| 2 | hsa-miR-206 | 11 | 0.003221 |
| 2 | hsa-miR-6864-3p | 3 | 0.004045 |
| 2 | hsa-miR-126-3p | 3 | 0.0055 |
| 2 | hsa-miR-3672 | 3 | 0.0055 |
| 2 | hsa-miR-205-3p | 4 | 0.00657 |
| 2 | hsa-miR-4645-3p | 3 | 0.007231 |
| 2 | hsa-miR-410-3p | 13 | 0.008238 |
| 2 | hsa-miR-7158-3p | 3 | 0.00925 |
| 3 | hsa-miR-215-5p | 10 | 5.47E-05 |
| 3 | hsa-miR-192-5p | 10 | 6.31E-05 |
| 3 | hsa-miR-193b-3p | 10 | 6.61E-05 |
| 3 | hsa-miR-515-5p | 5 | 0.000495 |
| 3 | hsa-miR-6871-5p | 3 | 0.001398 |
| 3 | hsa-miR-494-3p | 12 | 0.001423 |
| 3 | hsa-miR-8054 | 4 | 0.001832 |
| 3 | hsa-miR-548av-5p | 4 | 0.001832 |
| 3 | kshv-miR-K12-9-3p | 2 | 0.002041 |
| 3 | hsa-miR-548k | 4 | 0.00234 |
| 3 | hsa-miR-4521 | 3 | 0.003147 |
| 3 | hsa-miR-519e-5p | 4 | 0.003633 |
| 3 | hsa-miR-3139 | 3 | 0.005317 |
| 3 | hsa-miR-92a-3p | 8 | 0.0084 |
| 3 | hsa-miR-4714-3p | 2 | 0.008566 |
| 3 | hsa-miR-873-5p | 8 | 0.008805 |
| 3 | hsa-miR-2113 | 3 | 0.008872 |
