# Supplementary material for: Development of Multiscale Transcriptional Regulatory Network in Esophageal Cancer Based on Integrated Analysis
Source: Biomed Res Int. 2020 Aug 12;2020:5603958. doi: 10.1155/2020/5603958 (PMC7441423; doi:10.1155/2020/5603958)
Supplement: Supplementary Materials — Figure S1 (A) volcano plot of RNA-seq data between ESCA tissues and normal tissues. (B) Heatmap of DEGs with log2 (x +1) scale. Figure S2: (A) volcano plot of miRNA-seq data between ESCA tissues and normal tissues. (B) Heatmap of DEmiRNAs with log2 (x +1) scale. Figure S3: PPI network based on candidate gene set. Figure S4: heatmap showing the staging capabilities of key gene interaction modules. Figure S5: the diagnostic value of key regulators in distinguishing ESCA patients from normal controls based on GSE53625. (A) CXCL8: AUC 0.923. (B) KIF18A: AUC 0.899. (C) CYP2C8: AUC 0.922. (D) CYP4A11: AUC 0.738. (E) E2F1: AUC 0.849. Figure S6: the diagnostic value of key regulators in distinguishing ESCA patients at TNM I stage from normal controls based on GSE53625. (A) CXCL8: AUC 0.937. (B) CYP2C8: AUC 0.810. (C) E2F1: AUC 0.841. Supplementary Table 1: candidate gene interaction networks. Supplementary Table 2: pivot (ncRNA)-module pairs. Supplementary Table 3: pivot (TF)-module pairs. Supplementary Table 4: candidate regulators [file 5603958.f1.zip › Supplementary Table3.docx]

Supplementary Table3: Pivot (TF) - Module pairs

| Cluster | TF | Connection | P-value |
| --- | --- | --- | --- |
| 1 | NR1I3 | 5 | 2.25E-09 |
| 1 | AHR | 5 | 8.33E-06 |
| 1 | NR1I2 | 4 | 3.71E-05 |
| 1 | HNF4A | 4 | 5.49E-05 |
| 1 | STAT6 | 3 | 0.00074 |
| 1 | MBD2 | 2 | 0.005198 |
| 2 | RELA | 18 | 6.23E-06 |
| 2 | NFKB1 | 17 | 2.29E-05 |
| 2 | KLF2 | 2 | 0.007737 |
| 3 | E2F1 | 7 | 4.23E-05 |
| 3 | MED1 | 2 | 0.001454 |
