# Supplementary material for: Development of Multiscale Transcriptional Regulatory Network in Esophageal Cancer Based on Integrated Analysis
Source: Biomed Res Int. 2020 Aug 12;2020:5603958. doi: 10.1155/2020/5603958 (PMC7441423; doi:10.1155/2020/5603958)
Supplement: Supplementary Materials — Figure S1 (A) volcano plot of RNA-seq data between ESCA tissues and normal tissues. (B) Heatmap of DEGs with log2 (x +1) scale. Figure S2: (A) volcano plot of miRNA-seq data between ESCA tissues and normal tissues. (B) Heatmap of DEmiRNAs with log2 (x +1) scale. Figure S3: PPI network based on candidate gene set. Figure S4: heatmap showing the staging capabilities of key gene interaction modules. Figure S5: the diagnostic value of key regulators in distinguishing ESCA patients from normal controls based on GSE53625. (A) CXCL8: AUC 0.923. (B) KIF18A: AUC 0.899. (C) CYP2C8: AUC 0.922. (D) CYP4A11: AUC 0.738. (E) E2F1: AUC 0.849. Figure S6: the diagnostic value of key regulators in distinguishing ESCA patients at TNM I stage from normal controls based on GSE53625. (A) CXCL8: AUC 0.937. (B) CYP2C8: AUC 0.810. (C) E2F1: AUC 0.841. Supplementary Table 1: candidate gene interaction networks. Supplementary Table 2: pivot (ncRNA)-module pairs. Supplementary Table 3: pivot (TF)-module pairs. Supplementary Table 4: candidate regulators [file 5603958.f1.zip › Supplementary Table4.docx]

Supplementary Table4: Candidate regulators

| Group | Degree | Regulators |
| --- | --- | --- |
| Cluster2 | 36 | CXCL8 |
| ncRNA | 36 | hsa-miR-590-3p |
| Cluster2 | 33 | PTGER3 |
| Cluster2 | 33 | CXCL12 |
| Cluster1 | 31 | CYP2B6 |
| Cluster2 | 29 | CCR5 |
| Cluster2 | 29 | CXCL2 |
| Cluster2 | 28 | CNR1 |
| Cluster2 | 28 | CXCL1 |
| Cluster2 | 27 | CXCL5 |
| Cluster2 | 27 | CXCR2 |
| Cluster2 | 27 | CXCR4 |
| Cluster2 | 26 | CXCL11 |
| ncRNA | 26 | hsa-miR-494-3p |
| Cluster3 | 25 | MAD2L1 |
| Cluster3 | 25 | BIRC5 |
| Cluster2 | 24 | CCL20 |
| Cluster2 | 24 | RGS1 |
| Cluster3 | 24 | KIF18A |
| Cluster3 | 24 | BUB1B |
| ncRNA | 24 | hsa-miR-410-3p |
| Cluster3 | 23 | CENPA |
| Cluster3 | 23 | KIF2C |
| Cluster1 | 23 | CYP2C8 |
| Cluster2 | 23 | CXCL10 |
| Cluster2 | 22 | SAA1 |
| Cluster3 | 22 | CENPF |
| Cluster3 | 21 | CDC20 |
| Cluster1 | 21 | CYP4A11 |
| ncRNA | 21 | hsa-miR-1-3p |
| Cluster3 | 20 | AURKB |
| Cluster2 | 20 | CXCL6 |
| Cluster2 | 20 | P2RY14 |
| Cluster1 | 19 | CYP2C9 |
| Cluster1 | 19 | UGT1A1 |
| Cluster1 | 19 | CYP1A1 |
| Cluster2 | 19 | PPBP |
| Cluster1 | 19 | CYP2E1 |
| Cluster3 | 19 | CENPE |
| Cluster3 | 19 | NUF2 |
| Cluster3 | 19 | BUB1 |
| ncRNA | 19 | hsa-miR-92a-3p |
| Cluster2 | 18 | ANXA1 |
| Cluster3 | 18 | NDC80 |
| methy_low | 18 | NPY |
| Cluster3 | 18 | CDCA5 |
| Cluster1 | 17 | GSTP1 |
| ncRNA | 17 | hsa-miR-206 |
| Cluster3 | 16 | SKA1 |
| ncRNA | 16 | hsa-miR-873-5p |
| Cluster2 | 15 | CHRM2 |
| Cluster3 | 15 | AURKA |
| Cluster2 | 15 | CCL25 |
| pivot-TF | 15 | NFKB1 |
| pivot-TF | 15 | RELA |
| Cluster1 | 14 | AOX1 |
| Cluster2 | 14 | GPR17 |
| ncRNA | 14 | hsa-miR-192-5p |
| ncRNA | 14 | hsa-miR-215-5p |
| Cluster1 | 13 | ADH4 |
| Cluster1 | 13 | GSTM5 |
| Cluster1 | 13 | GSTM1 |
| Cluster1 | 12 | CYP26A1 |
| Cluster1 | 12 | ADH1B |
| methy_low | 12 | CASR |
| ncRNA | 12 | hsa-miR-193b-3p |
| Cluster3 | 11 | NEK2 |
| Cluster3 | 11 | PSMD14 |
| Cluster1 | 11 | ALDH1A1 |
| Cluster1 | 11 | EPHX1 |
| Cluster1 | 11 | ALOX15 |
| Cluster2 | 11 | PTGDR2 |
| Cluster2 | 10 | HTR1E |
| Cluster3 | 10 | DLGAP5 |
| Cluster3 | 10 | UBE2C |
| Cluster1 | 9 | ADH6 |
| Cluster2 | 8 | GPER1 |
| Cluster3 | 8 | SKA3 |
| Cluster1 | 7 | GPX7 |
| ncRNA | 7 | hsa-miR-515-5p |
| methy_low | 6 | DRD4 |
| methy_low | 6 | ALOX12B |
| methy_low | 6 | NR1I2 |
| ncRNA | 6 | hsa-miR-8054 |
| ncRNA | 6 | hsa-miR-548av-5p |
| ncRNA | 6 | hsa-miR-519e-5p |
| ncRNA | 6 | hsa-miR-548k |
| ncRNA | 5 | hsa-miR-4797-5p |
| ncRNA | 5 | hsa-miR-3160-5p |
| ncRNA | 5 | hsa-miR-7110-5p |
| methy_low | 4 | GNAQ |
| ncRNA | 4 | hsa-miR-205-3p |
| ncRNA | 4 | hsa-miR-6871-5p |
| ncRNA | 4 | hsa-miR-4645-3p |
| ncRNA | 4 | hsa-miR-6808-5p |
| ncRNA | 4 | hsa-miR-6842-5p |
| ncRNA | 4 | hsa-miR-2113 |
| ncRNA | 4 | hsa-miR-6752-5p |
| pivot-TF | 4 | E2F1 |
| pivot-TF | 4 | STAT6 |
| methy_low | 3 | GNA11 |
| ncRNA | 3 | hsa-miR-7158-3p |
| ncRNA | 3 | hsa-miR-126-3p |
| ncRNA | 3 | hsa-miR-4714-3p |
| ncRNA | 3 | hsa-miR-27b-5p |
| ncRNA | 3 | hsa-miR-4521 |
| ncRNA | 3 | hsa-miR-3672 |
| ncRNA | 3 | hsa-miR-3139 |
| ncRNA | 3 | hsa-miR-6880-5p |
| ncRNA | 3 | hsa-miR-6864-3p |
| ncRNA | 3 | hsa-miR-363-5p |
| ncRNA | 3 | hsa-miR-6745 |
| ncRNA | 3 | hsa-miR-1287-3p |
| ncRNA | 3 | hsa-miR-3665 |
| pivot-TF | 3 | AHR |
| pivot-TF | 3 | HNF4A |
| pivot-TF | 3 | NR1I3 |
| methy_low | 2 | MMP2 |
| methy_low | 2 | GALR3 |
| ncRNA | 2 | hsa-miR-3129-3p |
| ncRNA | 2 | hsa-miR-3165 |
| ncRNA | 2 | hsa-miR-5583-5p |
| ncRNA | 2 | kshv-miR-K12-9-3p |
| pivot-TF | 2 | KLF2 |
| pivot-TF | 2 | MED1 |
| methy_low | 1 | TRH |
| ncRNA | 1 | hsa-miR-99a-3p |
| pivot-TF | 1 | MBD2 |
