# Supplementary figures and images for: Development of Multiscale Transcriptional Regulatory Network in Esophageal Cancer Based on Integrated Analysis
Source: Biomed Res Int. 2020 Aug 12;2020:5603958. doi: 10.1155/2020/5603958 (PMC7441423; doi:10.1155/2020/5603958)

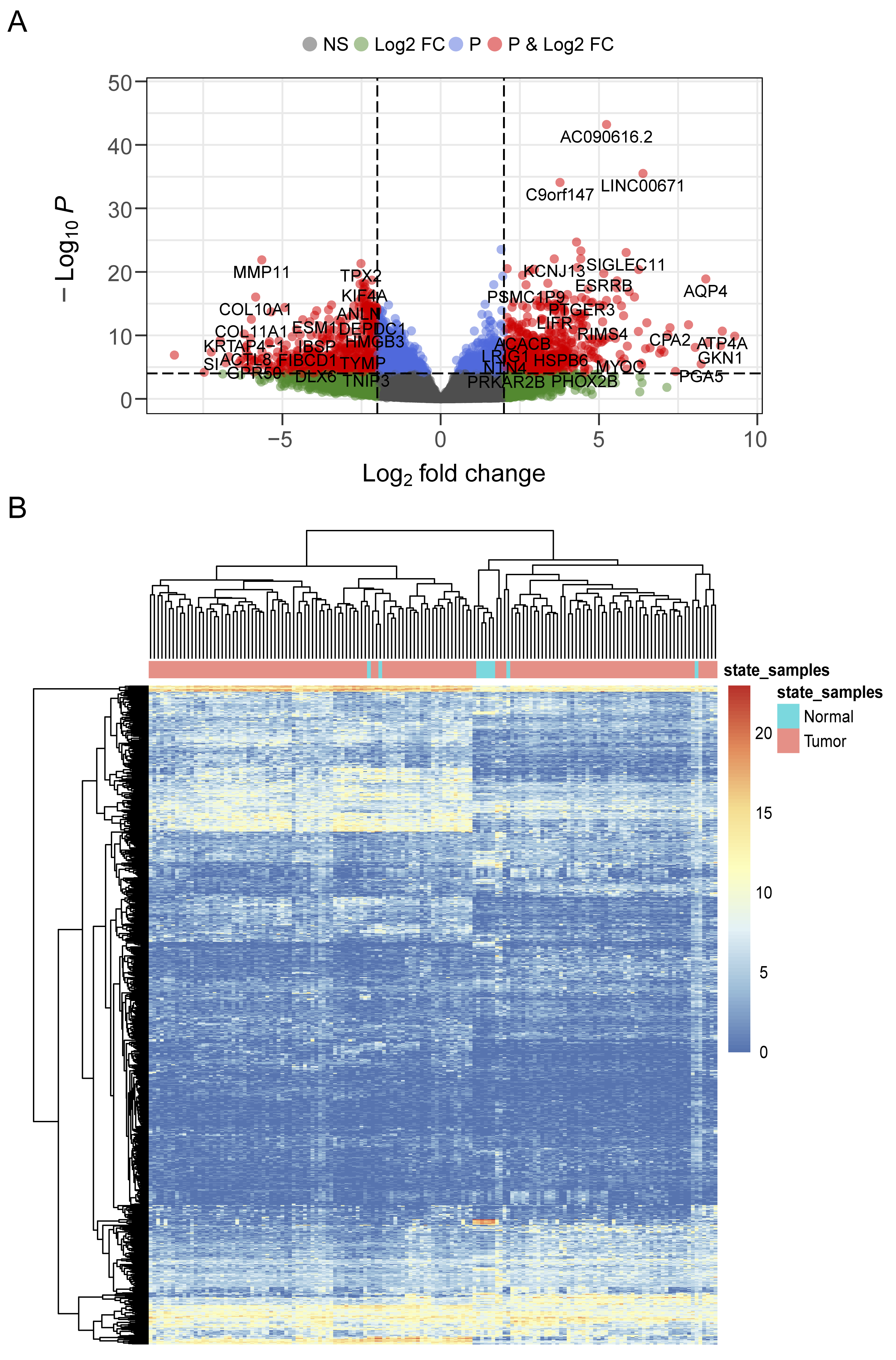

Supplement: Supplementary Materials — Figure S1 (A) volcano plot of RNA-seq data between ESCA tissues and normal tissues. (B) Heatmap of DEGs with log2 (x +1) scale. Figure S2: (A) volcano plot of miRNA-seq data between ESCA tissues and normal tissues. (B) Heatmap of DEmiRNAs with log2 (x +1) scale. Figure S3: PPI network based on candidate gene set. Figure S4: heatmap showing the staging capabilities of key gene interaction modules. Figure S5: the diagnostic value of key regulators in distinguishing ESCA patients from normal controls based on GSE53625. (A) CXCL8: AUC 0.923. (B) KIF18A: AUC 0.899. (C) CYP2C8: AUC 0.922. (D) CYP4A11: AUC 0.738. (E) E2F1: AUC 0.849. Figure S6: the diagnostic value of key regulators in distinguishing ESCA patients at TNM I stage from normal controls based on GSE53625. (A) CXCL8: AUC 0.937. (B) CYP2C8: AUC 0.810. (C) E2F1: AUC 0.841. Supplementary Table 1: candidate gene interaction networks. Supplementary Table 2: pivot (ncRNA)-module pairs. Supplementary Table 3: pivot (TF)-module pairs. Supplementary Table 4: candidate regulators [file 5603958.f1.zip › Figure S1.tif]

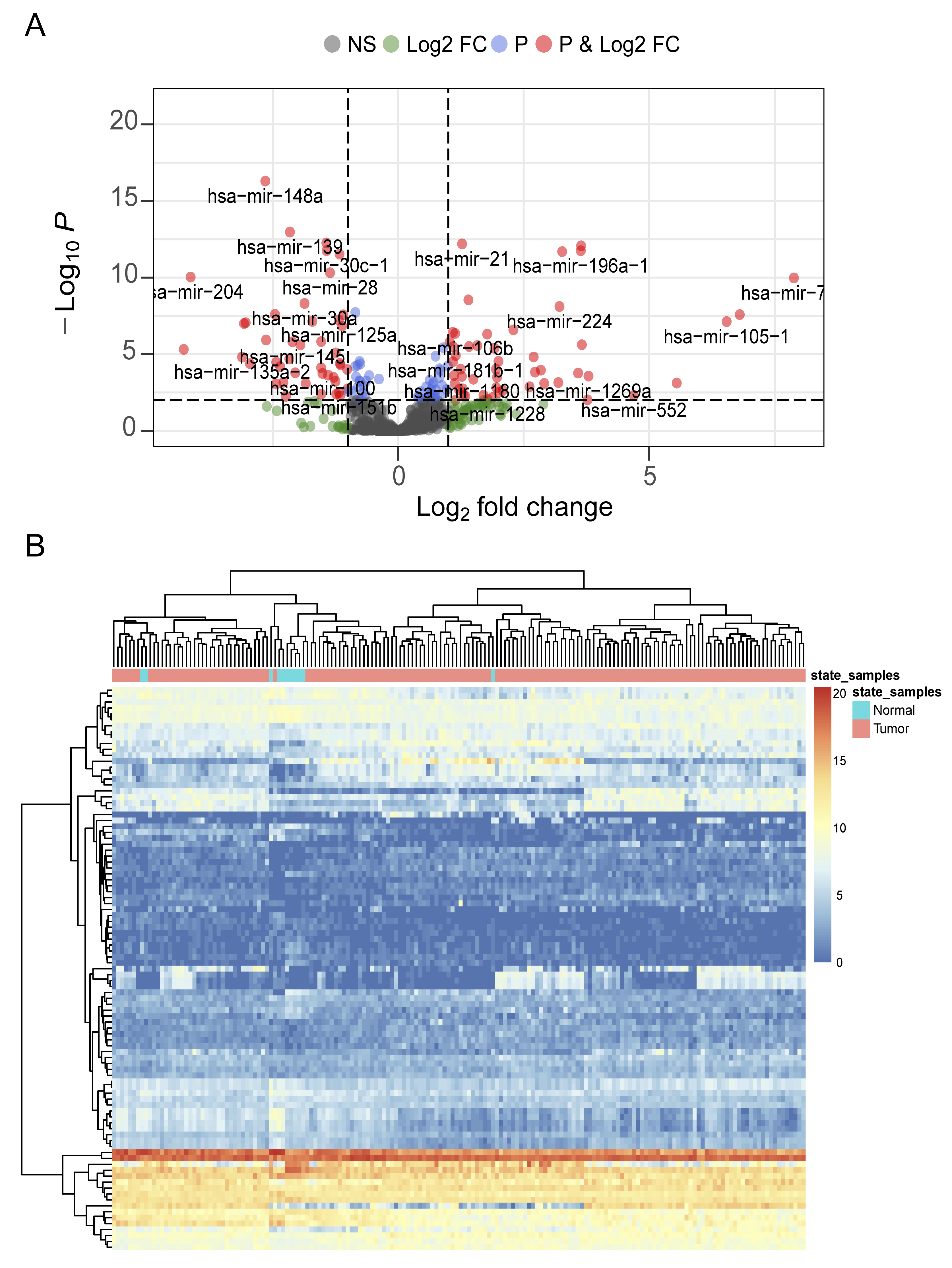

Supplement: Supplementary Materials — Figure S1 (A) volcano plot of RNA-seq data between ESCA tissues and normal tissues. (B) Heatmap of DEGs with log2 (x +1) scale. Figure S2: (A) volcano plot of miRNA-seq data between ESCA tissues and normal tissues. (B) Heatmap of DEmiRNAs with log2 (x +1) scale. Figure S3: PPI network based on candidate gene set. Figure S4: heatmap showing the staging capabilities of key gene interaction modules. Figure S5: the diagnostic value of key regulators in distinguishing ESCA patients from normal controls based on GSE53625. (A) CXCL8: AUC 0.923. (B) KIF18A: AUC 0.899. (C) CYP2C8: AUC 0.922. (D) CYP4A11: AUC 0.738. (E) E2F1: AUC 0.849. Figure S6: the diagnostic value of key regulators in distinguishing ESCA patients at TNM I stage from normal controls based on GSE53625. (A) CXCL8: AUC 0.937. (B) CYP2C8: AUC 0.810. (C) E2F1: AUC 0.841. Supplementary Table 1: candidate gene interaction networks. Supplementary Table 2: pivot (ncRNA)-module pairs. Supplementary Table 3: pivot (TF)-module pairs. Supplementary Table 4: candidate regulators [file 5603958.f1.zip › Figure S2.tif]

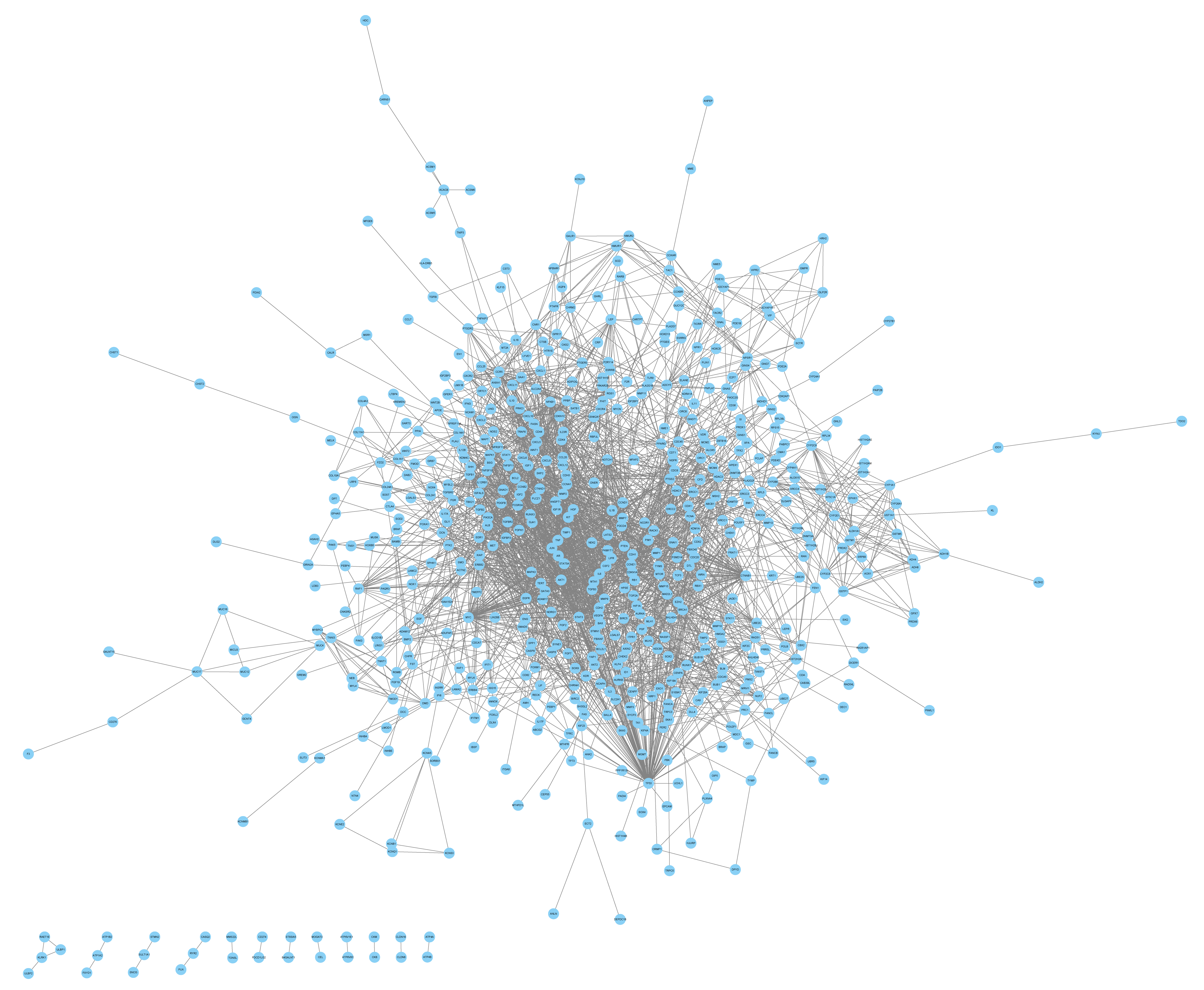

Supplement: Supplementary Materials — Figure S1 (A) volcano plot of RNA-seq data between ESCA tissues and normal tissues. (B) Heatmap of DEGs with log2 (x +1) scale. Figure S2: (A) volcano plot of miRNA-seq data between ESCA tissues and normal tissues. (B) Heatmap of DEmiRNAs with log2 (x +1) scale. Figure S3: PPI network based on candidate gene set. Figure S4: heatmap showing the staging capabilities of key gene interaction modules. Figure S5: the diagnostic value of key regulators in distinguishing ESCA patients from normal controls based on GSE53625. (A) CXCL8: AUC 0.923. (B) KIF18A: AUC 0.899. (C) CYP2C8: AUC 0.922. (D) CYP4A11: AUC 0.738. (E) E2F1: AUC 0.849. Figure S6: the diagnostic value of key regulators in distinguishing ESCA patients at TNM I stage from normal controls based on GSE53625. (A) CXCL8: AUC 0.937. (B) CYP2C8: AUC 0.810. (C) E2F1: AUC 0.841. Supplementary Table 1: candidate gene interaction networks. Supplementary Table 2: pivot (ncRNA)-module pairs. Supplementary Table 3: pivot (TF)-module pairs. Supplementary Table 4: candidate regulators [file 5603958.f1.zip › Figure S3.tif]

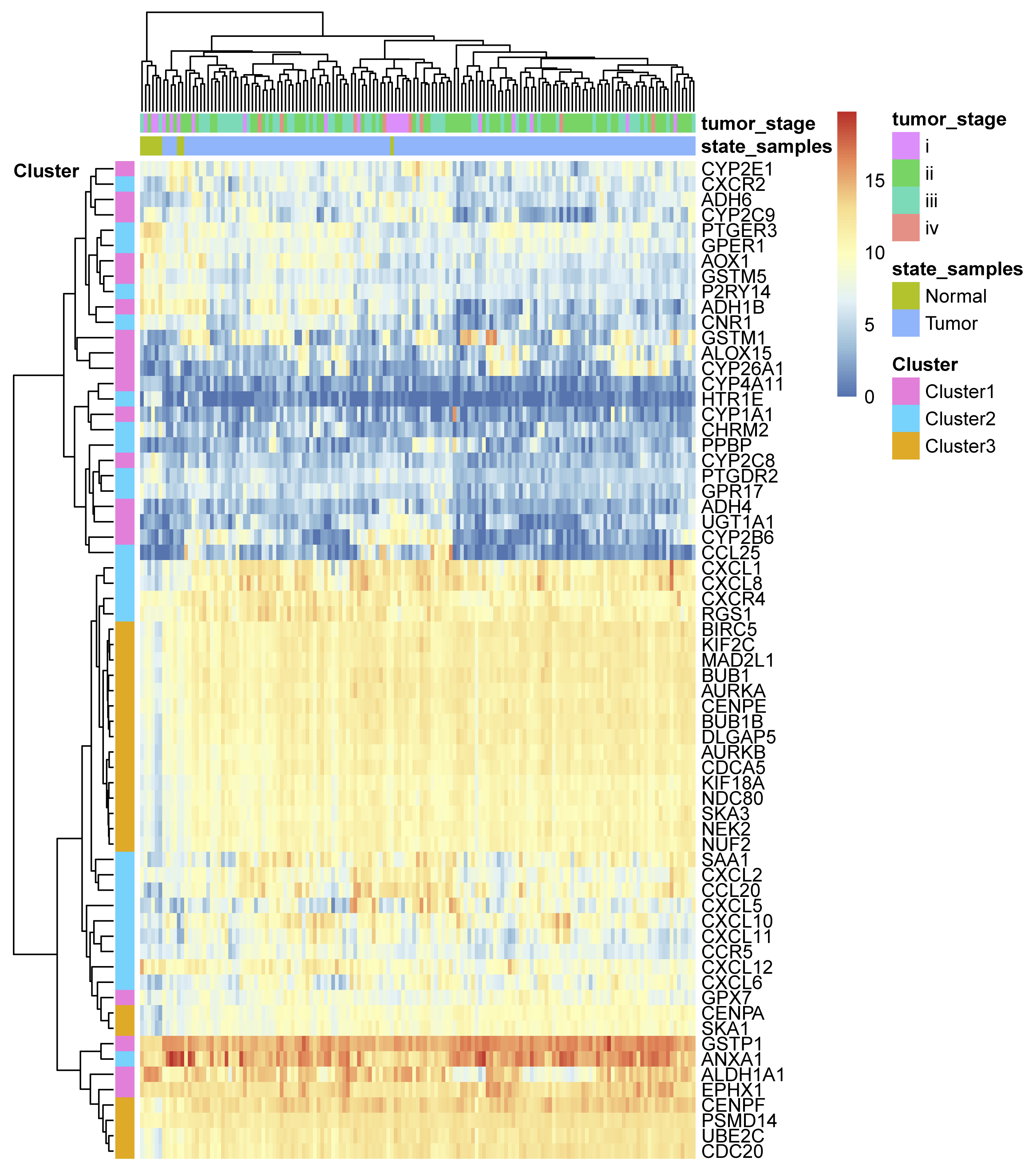

Supplement: Supplementary Materials — Figure S1 (A) volcano plot of RNA-seq data between ESCA tissues and normal tissues. (B) Heatmap of DEGs with log2 (x +1) scale. Figure S2: (A) volcano plot of miRNA-seq data between ESCA tissues and normal tissues. (B) Heatmap of DEmiRNAs with log2 (x +1) scale. Figure S3: PPI network based on candidate gene set. Figure S4: heatmap showing the staging capabilities of key gene interaction modules. Figure S5: the diagnostic value of key regulators in distinguishing ESCA patients from normal controls based on GSE53625. (A) CXCL8: AUC 0.923. (B) KIF18A: AUC 0.899. (C) CYP2C8: AUC 0.922. (D) CYP4A11: AUC 0.738. (E) E2F1: AUC 0.849. Figure S6: the diagnostic value of key regulators in distinguishing ESCA patients at TNM I stage from normal controls based on GSE53625. (A) CXCL8: AUC 0.937. (B) CYP2C8: AUC 0.810. (C) E2F1: AUC 0.841. Supplementary Table 1: candidate gene interaction networks. Supplementary Table 2: pivot (ncRNA)-module pairs. Supplementary Table 3: pivot (TF)-module pairs. Supplementary Table 4: candidate regulators [file 5603958.f1.zip › Figure S4.tif]

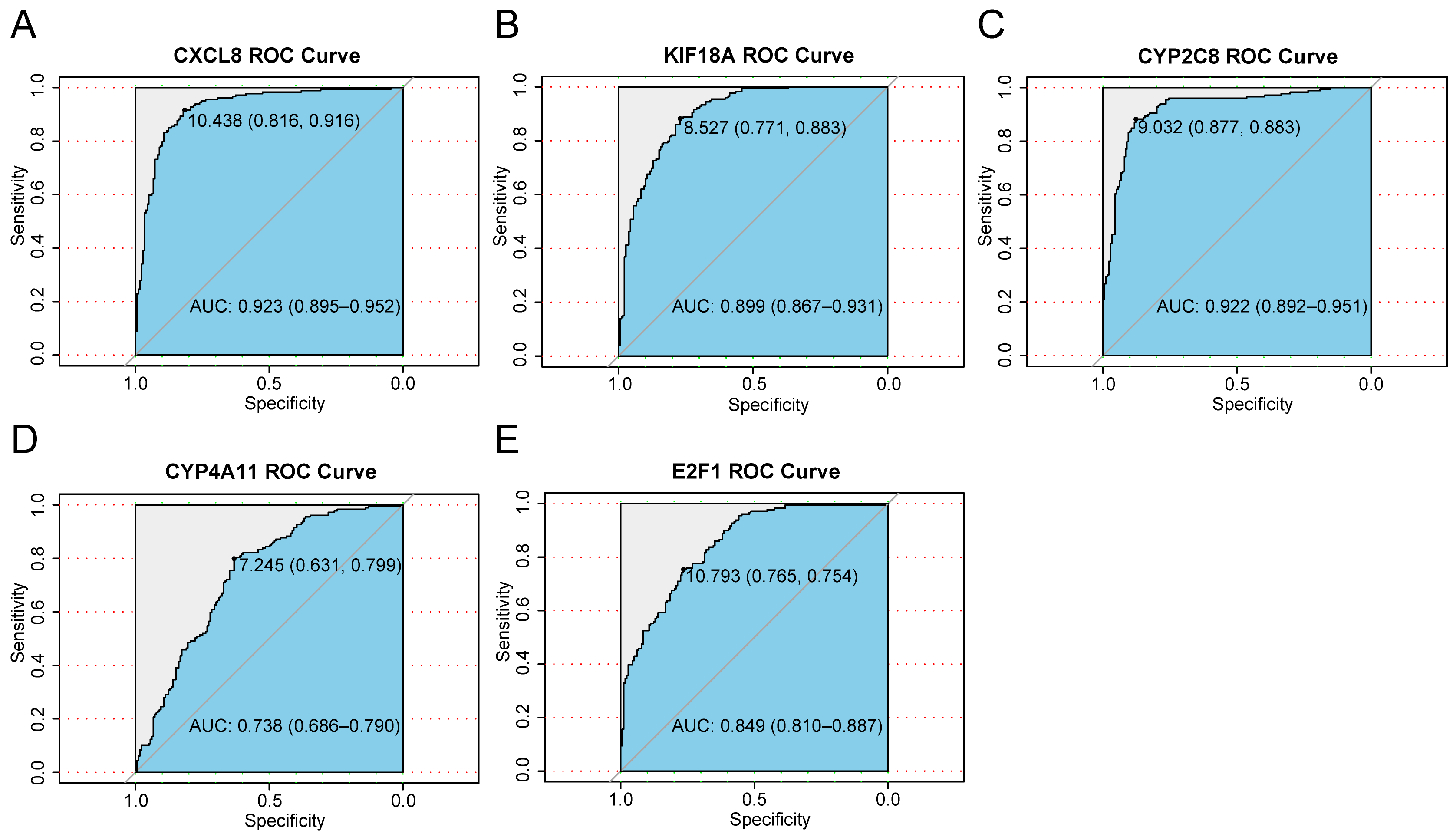

Supplement: Supplementary Materials — Figure S1 (A) volcano plot of RNA-seq data between ESCA tissues and normal tissues. (B) Heatmap of DEGs with log2 (x +1) scale. Figure S2: (A) volcano plot of miRNA-seq data between ESCA tissues and normal tissues. (B) Heatmap of DEmiRNAs with log2 (x +1) scale. Figure S3: PPI network based on candidate gene set. Figure S4: heatmap showing the staging capabilities of key gene interaction modules. Figure S5: the diagnostic value of key regulators in distinguishing ESCA patients from normal controls based on GSE53625. (A) CXCL8: AUC 0.923. (B) KIF18A: AUC 0.899. (C) CYP2C8: AUC 0.922. (D) CYP4A11: AUC 0.738. (E) E2F1: AUC 0.849. Figure S6: the diagnostic value of key regulators in distinguishing ESCA patients at TNM I stage from normal controls based on GSE53625. (A) CXCL8: AUC 0.937. (B) CYP2C8: AUC 0.810. (C) E2F1: AUC 0.841. Supplementary Table 1: candidate gene interaction networks. Supplementary Table 2: pivot (ncRNA)-module pairs. Supplementary Table 3: pivot (TF)-module pairs. Supplementary Table 4: candidate regulators [file 5603958.f1.zip › Figure S5.tif]

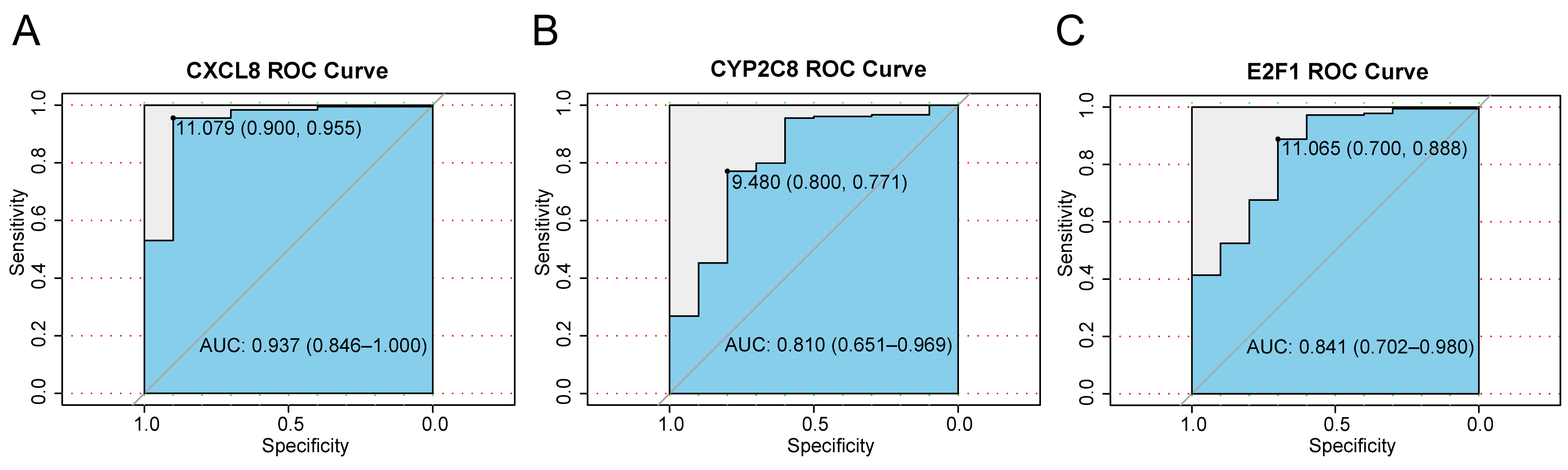

Supplement: Supplementary Materials — Figure S1 (A) volcano plot of RNA-seq data between ESCA tissues and normal tissues. (B) Heatmap of DEGs with log2 (x +1) scale. Figure S2: (A) volcano plot of miRNA-seq data between ESCA tissues and normal tissues. (B) Heatmap of DEmiRNAs with log2 (x +1) scale. Figure S3: PPI network based on candidate gene set. Figure S4: heatmap showing the staging capabilities of key gene interaction modules. Figure S5: the diagnostic value of key regulators in distinguishing ESCA patients from normal controls based on GSE53625. (A) CXCL8: AUC 0.923. (B) KIF18A: AUC 0.899. (C) CYP2C8: AUC 0.922. (D) CYP4A11: AUC 0.738. (E) E2F1: AUC 0.849. Figure S6: the diagnostic value of key regulators in distinguishing ESCA patients at TNM I stage from normal controls based on GSE53625. (A) CXCL8: AUC 0.937. (B) CYP2C8: AUC 0.810. (C) E2F1: AUC 0.841. Supplementary Table 1: candidate gene interaction networks. Supplementary Table 2: pivot (ncRNA)-module pairs. Supplementary Table 3: pivot (TF)-module pairs. Supplementary Table 4: candidate regulators [file 5603958.f1.zip › Figure S6.tif]
